# Bilateral electrical pudendal nerve stimulation as additional therapy for lower urinary tract dysfunction when stage II sacral neuromodulator fails: a case report

**DOI:** 10.1186/s12894-021-00808-5

**Published:** 2021-03-10

**Authors:** Shan Chen, Siyou Wang, Yunqiu Gao, Xiaolian Lu, Jiasheng Yan, Lihua Xuan, Shenhong Wang

**Affiliations:** 1 Department of Acupuncture and Moxibustion, The First Affiliated Hospital of Zhejiang Chinese Medical University, Hangzhou, China; 2 Clinical Research Section, Shanghai Research Institute of Acupuncture and Meridian, Shanghai, China; 3 Department of Urology, The First Affiliated Hospital of Zhejiang Chinese Medical University, No. 54 Youdian Road, Hangzhou, 310006 Zhejiang Province China

**Keywords:** Sacral modulation, Lower urinary tract dysfunction, Electrical pudendal nerve stimulation, Failure, Case report

## Abstract

**Background:**

Sacral neuromodulation (SNM) has become an effective therapy for patients with lower urinary tract dysfunction (LUTD) who do not respond to conservative treatment. However, an effective treatment strategy for patients who fail SNM has not yet been identified. An option for LUTD is needed when the clinical response to the SNM diminishes.

**Case presentation:**

A 51-year-old Chinese man presented to an outpatient clinic complaining of difficulty in urination for > 3 years. The patient also complained of urinary frequency and urgency, accompanied by perineal discomfort. He was diagnosed with LUTD based on his symptoms and previous examinations. The patient underwent sacral neuromodulation with a permanent implantable pulse generator (IPG) (provided free of charge by Chengnuo Medical Technology Co., Ltd.; General Stim, Hangzhou, China) in the left buttock, as he participated in the company’s clinical trial to test the long-term effects of IPG. He reported loss of efficacy of the device 3 months after the implantation. We performed bilateral electrical pudendal nerve stimulation (EPNS) therapy for him. After 2 weeks of treatment, he began to report smooth voiding within 2 h after EPNS, and a moderate improvement in urinary frequency, urgency, and perineal discomfort. After 4 weeks of EPNS, the patient reported > 50% improvement in his urination, evaluated with the short form of the International Consultation on Incontinence Questionnaire for Male Lower Urinary Tract Symptoms. He reported smooth voiding, moderate improvements in urinary frequency and urgency, and the disappearance of the perineal discomfort. He also reported improved sleep and erections. The patient was discharged after 8 weeks of EPNS treatment.

**Conclusion:**

EPNS could be an option as an additional therapy for patients with LUTD who have failed SNM.

**Supplementary Information:**

The online version contains supplementary material available at 10.1186/s12894-021-00808-5.

## Background

Lower urinary tract dysfunction (LUTD) includes a broad spectrum of diseases ranging from failure to empty the bladder to failure to store urine [1]. Sacral neuromodulation (SNM) using InterStim is a minimally invasive therapy approved by the United States Food and Drug Administration for LUTD [2]. Over the last 20 years, SNM has become an effective therapy used in China for patients with LUTD who do not respond to conservative treatment [3]. However, one-third of patients required reoperation, often due to a lack of efficacy or worsening symptoms [4]. This case report is the first to use electrical pudendal nerve stimulation (EPNS) as an additional therapy for a patient who did not respond to stage II sacral neuromodulation.

## Case presentation

**Fig. 1:**
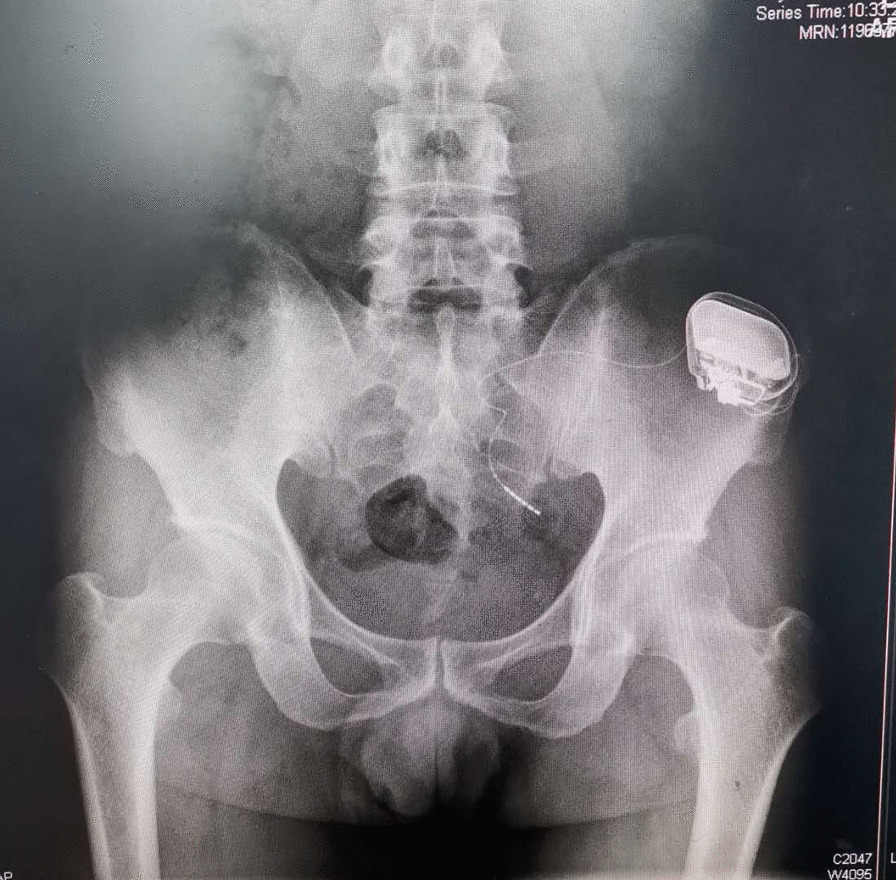
The radiograph of the implantable pulse generator in the patient’s left buttock

A 51-year-old Chinese man (height, 168 cm; weight, 59 kg) presented to an outpatient clinic complaining of difficulty urination for > 3 years. He described urinary hesitancy for 2–3 min, urinary straining, and a weak urinary stream. The patient also had urinary retention 1–3 times per week for over 2 years, for which he performed intermittent self-catheterisation with around 120 mL post-void residual urine volume according to the urethral catheter output. The patient also complained of both daytime (12–14 times) and nocturnal (4–5 times) urinary frequencies and an urgency with 50–200 mL urine volume every time depending on the amount of fluid intake accompanied by perineal discomfort since he had an episode of withholding urination approximately 2 years prior. The patient also had erectile dysfunction. He had presented to multiple urology clinics over the last 3 years, undergoing multiple diagnostic examinations, including urinalysis, urinary ultrasound, urodynamic studies, cystoscope, pelvic floor muscle electromyography (PFM EMG), and magnetic resonance imaging (MRI) of the lumbar vertebrae to the sacrococcygeal vertebrae. The urinalysis result was negative. Urinary ultrasound revealed a prostate of normal size with < 30 mL of post-void residual urine volume; however, prostatic calcification was noted. Urodynamics showed an underactive detrusor but no obstruction (Table 1). Cystoscopy showed lack of any anatomical obstruction and a normal bladder. PFM EMG indicated an abnormal bulbocavernosus reflex, and no abnormalities were detected on the pudendal nerve (PN) somatosensory evoked potential. No neurologic or urinary abnormalities were observed on MRI. Based on his symptoms and previous examinations, he was diagnosed with LUTD with the sub-category of overactive bladder (OAB) syndrome. The patient was prescribed tamsulosin hydrochloride (0.2 mg daily) and baclofen (10 mg daily); however, these medications were discontinued due to ineffectiveness. SNM was presented as an option to the patient more than 1 year ago. Following the first stage of SNM, the patient reported mild improvement. He then received a permanent implantable pulse generator (IPG) (Stage II) in the left buttock (Fig. 1), which was provided free of charge by Chengnuo Medical Technology Co., Ltd., (General Stim, Hangzhou, China), as he participated in the company’s clinical trial to test the long-term effects of IPG. However, he reported no further improvement after the implantation with a loss of efficacy of the device 3 months later, and he also felt irritable when the IPG was turned on. The patient also complained of insomnia and depression.

**Table 1:** Urodynamic study results

First sensation	210 mL
First desire to void	320 mL
Strong desire to void	360 mL
P detQmax	32 cm H_2_O
Maximum flow rate (Qmax)	11 mL/s
Residual urine	10 mL
Rectal tone	Normal

*P det* pressure of the detrusor, *Qmax* the maximum flow rate

**Fig. 2:**
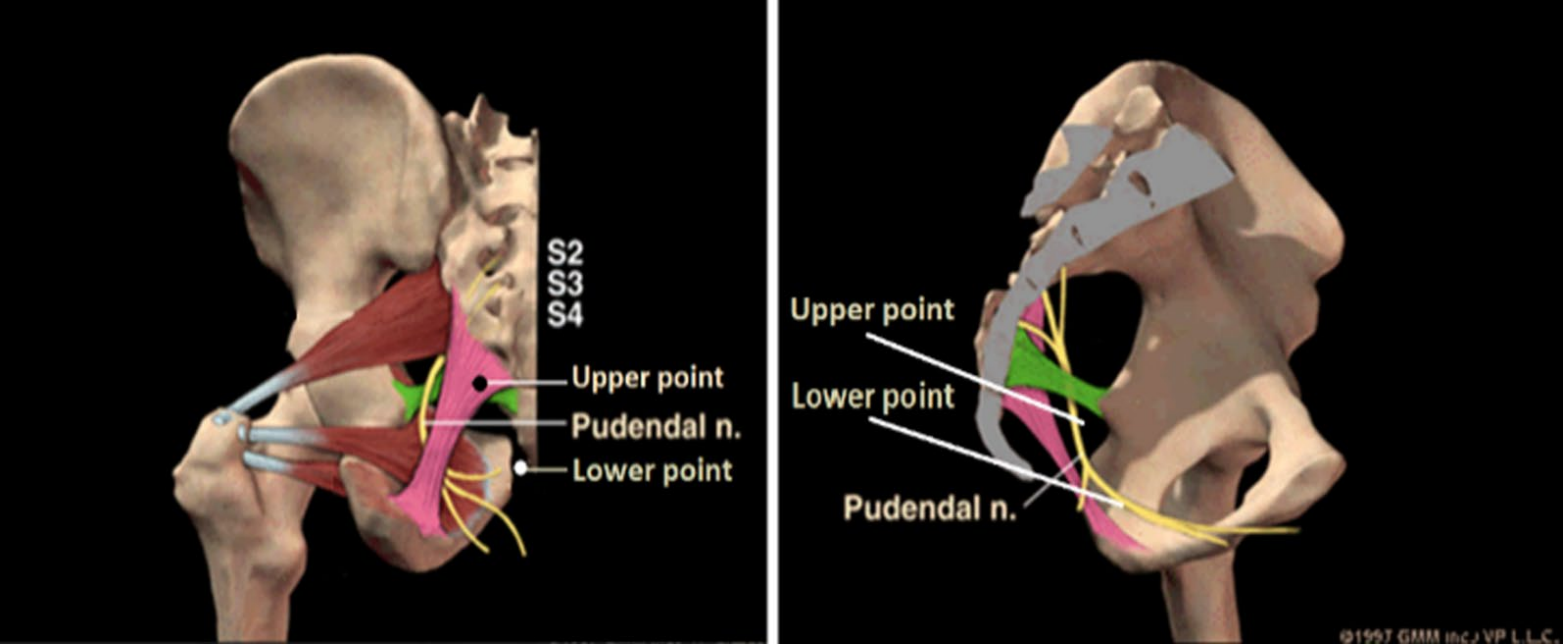
The anatomical locations of the four sacral points for electrostimulation: the four points used for the electrostimulation in this patient are shown. The two upper points are located approximately 1 cm from either side of the sacrococcygeal joint, and the two lower points are located approximately 1 cm from either side of the tip of the coccyx

The patient presented to our clinic > 1 year after the IPG was implanted due to its ineffectiveness and adverse effects. We agreed with the clinical diagnosis of LUTD and OAB syndrome [5] and performed bilateral EPNS therapy for him. Four sacral points were selected (Fig. 2) [6]. The two upper points were located approximately 1 cm on either side of the sacrococcygeal joint, and the two lower points were located approximately 1 cm on either side of the tip of the coccyx. At the upper points, a needle (Suzhou Shenlong Medical Apparatus Factory, Suzhou, China) of 0.40 × 100 mm was inserted perpendicularly to a depth of 80 mm to induce a sensation referred to the urethra or anus via stimulation of the main trunk of the PN. At the lower points, a needle of 0.40 × 100 mm was inserted obliquely toward the ischiorectal fossa to a depth of 90 mm to induce a sensation referred to the urethra via stimulation of the perineal nerve. Each pair of the ipsilaterally inserted needles were connected to two pairs of electrodes from a G6805-Aelectroacupuncture device (Shantou Medical Equipment Factory, Shantou, China), with the anode connected to the upper needle and the cathode connected to the lower needle [7, 8]. The device was set to produce electrical stimulation (biphasic 2-ms pulse) at a frequency of 2.0 Hz and a moderate intensity of 25–35 mA. Electrostimulation was performed for 60 min during each treatment. PFM contraction around the urethra was maintained during the entire electrostimulation procedure. The electrostimulation procedure was performed once daily, Monday through Friday. The patient’s IPG was turned off during the procedure. During treatments, the patient was hospitalised in the acupuncture inpatient department because he lived 200 km from the clinic. After 2 weeks of treatment, he began to report smooth voiding within 2 h after EPNS and a moderate improvement in urinary frequency, urgency, and discomfort. At this time, the patient began turning off his IPG for 2–6 h instead of only during EPNS. Two weeks later, after 4 weeks of EPNS, the patient reported > 50% improvement in his urination and stopped using the IPG. He reported smooth voiding and moderate improvements of urinary frequency and urgency and a disappearance of perineal discomfort. He also reported improved sleep and erections. He was discharged after 8 weeks of EPNS treatment. The patient was evaluated with the short form of the International Consultation on Incontinence Questionnaire for Male Lower Urinary Tract Symptoms (ICIQ-MLUTS). ICIQ-MLUTS contains 13 questions on the patient’s symptoms including urinary hesitancy, urinary straining, strength of urinary stream, interrupted urination, feeling of incomplete emptying, urinary urgency, urgency urinary incontinence, stress urinary incontinence, urinary incontinence for no obvious reason, enuresis, urine dribbling, urinary frequency, and nocturia. Every question was scored with 0–4 points, with a higher score indicating more severe symptoms, and the total scores ranged from 0 to 52 points. Each question had a sub-question where the patient rated the symptom’s impact on quality of life from 0 to 10 points; this did not count towards the total score. The patient scored 30 points before our treatment, and scored 22, 14, and 13 points after 2, 4, and 8 weeks of EPNS therapy, respectively (Additional file 1: Table S1). He achieved > 50% improvement after 4 weeks of EPNS therapy based on the results of the ICIQ-MLUTS. The patient also indicated a reduction in the impact of the symptoms, suggesting a better quality of life.

The patient was followed up via a phone interview 1 month later, during which he reported that he experienced smooth voiding for the first 2 weeks after the EPNS was discontinued. However, in the third and fourth week, his voiding sometimes became difficult. He had no choice at home but to turn on the IPG again, which he thought might help his voiding. His voiding difficulty persisted, but to a lower degree. Other symptoms such as urinary frequency and urgency remained moderately improved. He experienced a slight discomfort around his perineum. The patient expressed interest in continuing EPNS treatment in the future. He is being followed-up via telephone at 1-month intervals.

## Discussion and conclusion

SNMs have become increasingly popular in the treatment of LUTD in China in recent years [9]. However, investigations after SNM implantation regarding patients’ long-term efficacy or satisfaction has received little attention [10]. A survey conducted at Toronto Western Hospital involving 71 patients who had undergone SNM implantation found that the patients’ satisfaction with SNM was correlated with the need for additional medications for symptom control. Twenty-two (31%) of the patients used pain medication, intermittent self-catheterisation, intravesical Botox injections, anticholinergics, or combined mediations [11]. Additional therapy for LUTD was necessary after the clinical response to the SNM diminished.

The PN is a peripheral nerve with sympathetic fibres from the second, third, and fourth sacral nerve roots and motor and sensory functions [12]. The main trunk of the PN passes between the sacrotuberous and sacrospinous ligaments and enters Alcock’s canal in the ischiorectal fossa. Within the fossa, the PN branches into the perineal nerve that innervates the PFM and the skin of the labium majus as well as the dorsal nerve of the clitoris that innervates the skin of the clitoral shaft [13]. Because SNM therapy stimulates only some of the afferent fibres of the pudendal nerve, direct neurostimulation of the PN may be more effective [14, 15]. Pudendal neurostimulation (PNM) is growing as an alternative surgical option for patients who experience a decrease in the effectiveness of SNM and in those who were never satisfied after an SNM procedure [16, 17]. The PNM procedure involves a small implantable neurostimulator device (Bion, Advanced Bionics Corporation, Valencia, CA, USA) placed directly into one side of Alcock’s canal to stimulate the PN [15, 18]. The disadvantages of PNM are similar to those of SNM and include invasiveness, high cost of treatment, high surgical revision rate, device replacement when the battery runs out, and adverse events such as pain and infection [19].

**Fig. 3:**
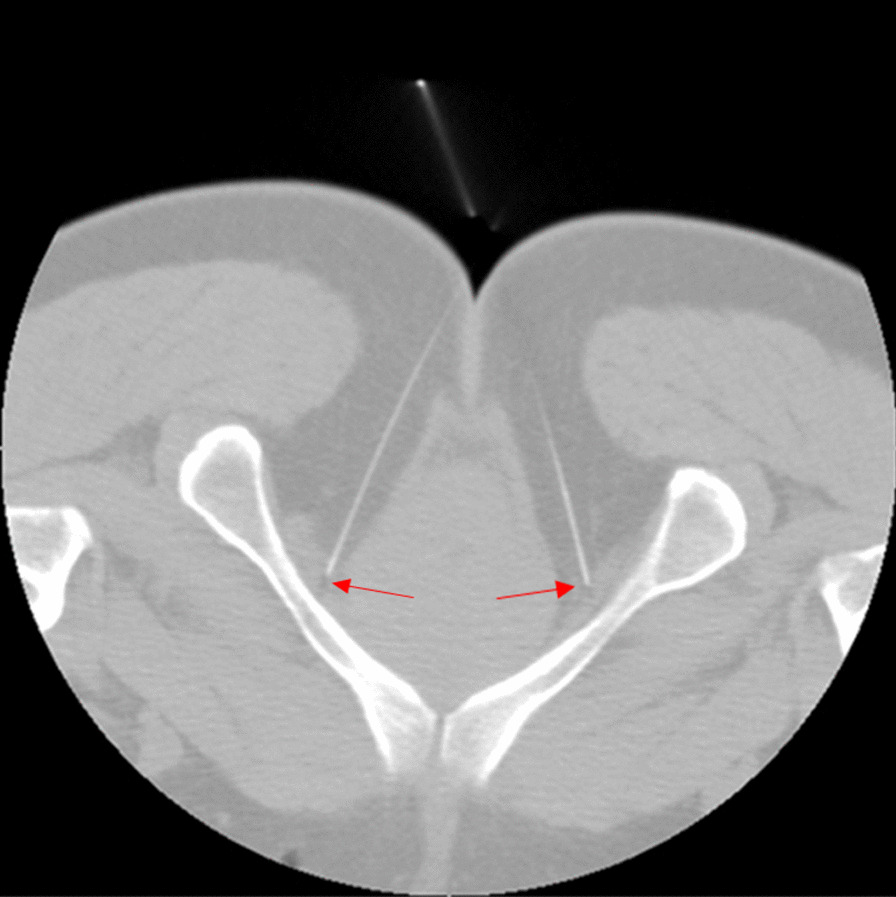
Transverse computed tomography (CT) scan of the coccygeal apex: A transverse CT image of the coccygeal apex shows the tips of needles inserted at the lower sacral point in the ischiorectal fossa (adjacent to the pudendal nerve in Alcock’s canal)

In EPNS therapy, four long needles are inserted at four points near the sacrococcyx in order to stimulate the PN within the sacrococcygeal region [20]. We have previously reported that the tip of a long acupuncture needle can reach Alcock’s canal where the perineal branch of the PN is located (Fig. 3) [13]. EPNS has been shown to cause PN excitation when used to treat stress urinary incontinence in women [21]. Additionally, EPNS has been used to treat female urgency-frequency syndrome and idiopathic urgency urinary incontinence [14]. A pilot study of the efficacy of EPNS on neurogenic lower urinary tract diseases compared with that of anogenital electrical stimulation found that EPNS is more effective than anogenital electrical stimulation in the short term [22].

In this case, the patient had only mild improvement (< 50% improvement in symptoms) after Stage I. In clinical practice, clinical symptom improvement ≤ 50% was considered to indicate poor efficacy, and these patients were recommended for other treatment [3].However, the patient accepted Stage II permanent implantation as part of the company’s clinical trial to test the long-term effects of IPG. Unfortunately, he failed the test, and his mood was substantially impaired because of voiding difficulty as well as urinary urgency and frequency symptoms. He described his life status as ‘desperate’ when he first presented to the author’s clinic. This case is an example of the use of EPNS as an additional therapy for LUTD when SNM is no longer clinically effective.

However his Bladder Outlet Obstruction Index (BOOI) was 10 and Bladder Contractility Index (BCI) was 87 consistent with an underactive detrusor without any obstruction. An underactive detrusor has been defined as BCI less than 100 while obstruction has been defined as BOOI more than 40 [23]. Additionally, cystoscopy failed to demonstrate any anatomical obstruction. Therefore we diagnosed the patient with detrusor underactivity as a sub-category under LUTD.

Other differential diagnoses of this patient included benign prostatic hyperplasia, urethral stricture and bladder pain syndrome. The ultrasound revealed a prostate of normal size; thus, his lower urinary tract symptoms were not secondary to benign prostatic hyperplasia. The aetiology of urethral stricture usually involves trauma due to traffic injuries or iatrogenic causes such as late failure of surgery for hypospadia or a stricture resulting from endoscopic manipulation [24]. Urethral stricture has other signs and symptoms including urinary tract infection, epididymitis, rising post-void residual urine volume, or decreased force of ejaculation [24]. The patient had neither history of trauma, urinary surgeries, nor other symptoms including urinary tract infection or epididymitis, so urethral stricture was excluded. Diagnostic criteria for bladder pain syndrome (also known as interstitial cystitis) [25] include unpleasant symptoms associated with bladder pain (usually suprapubic) or pressure, where the pain or pressure typically increases with increased bladder volume; and other lower urinary symptoms (e.g., urinary frequency) present for at least 6 weeks and no other causes of symptoms. The patient in our case has no suprapubic pain or pressure associated with the bladder, though he had urinary frequency over 6 weeks. His perineal discomfort was not painful and stayed only at the perineum and did not increase with bladder filling. Besides, his cystoscope report was clear without finding Hunner’s lesions. Since the patient did not meet the first major diagnostic criteria and cystoscope had ruled out Hunner’s lesions of the bladder, he was not diagnosed with bladder pain syndrome. In summary, the diagnosis of this patient was LUTD with detrusor underactivity and OAB syndrome as sub-categories.

Regarding pharmacotherapy, the patient was prescribed 0.2 mg of tamsulosin before the EPNS treatment. Tamsulosin is a first-line treatment for male LUTD. The literature recommends 0.2 mg of tamsulosin as the initial dose for Asian men with LUTD [26]. Unfortunately, he was refractory to tamsulosin and terminated its use. Further, the main reason for his voiding difficulty was due to underactive detrusor. In our view, tamsulosin was ineffective because it helps to relax the prostatic urethra and bladder neck through alpha-adrenergic blockade. The patient was not prescribed antimuscarinics, which are used as first-line pharmacological therapy for OAB by his previous urological doctor who considered that antimuscarinics might aggravate his urinary retention through side effects of this medicine [27].

This report is not without limitations. As a case report of a single patient, the study has no statistical power. The long-term effects of EPNS was also not studied. Prospective, randomised studies with large sample sizes are required to investigate the efficacy and safety of EPNS in patients with LUTD.

In conclusion, EPNS could be an option as an additional therapy for LUTD patients who do not respond to SNM.

## Supplementary Information

**Additional file 1: Supplymentary Table 1**. Lower urinary tract symptoms changes and bothersomeness index at each point of EPNS therapy investgated by Short form of International Consultation on Incontinence Questionnaire - Male Lower Urinary Tract Symptoms (ICIQ-MLUTS).

## Data Availability

All data generated or analysed during this study are included in this published article.

## Abbreviations

LUTD: Lower urinary tract dysfunction
SNM: Sacral neuromodulation
EPNS: Electrical pudendal nerve stimulation
PFM EMG: Pelvic floor muscle electromyography
MRI: Magnetic resonance imaging
ICIQ-MLUTS: International Consultation on Incontinence Questionnaire for Male Lower Urinary Tract Symptoms
PN: Pudendal nerve
SEP: Somatosensory evoked potential
IPG: Implantable pulse generator
PNM: Pudendal neuromodulation
BOOI: Bladder Outlet Obstruction Index
BCI: Bladder Contractility Index
OAB: Overactive bladder
