# Supplementary material for: Bilateral electrical pudendal nerve stimulation as additional therapy for lower urinary tract dysfunction when stage II sacral neuromodulator fails: a case report
Source: BMC Urol. 2021 Mar 10;21:37. doi: 10.1186/s12894-021-00808-5 (PMC7945661; doi:10.1186/s12894-021-00808-5)
Supplement: Supplementary file 1 — Additional file 1: Supplymentary Table 1. Lower urinary tract symptoms changes and bothersomeness index at each point of EPNS therapy investgated by Short form of International Consultation on Incontinence Questionnaire - Male Lower Urinary Tract Symptoms (ICIQ-MLUTS). [file 12894_2021_808_MOESM1_ESM.docx]

Supplementary Table 1 The score of the patient at each endpoint of the therapy and quality of life index based on the short form of the ICIQ-MLUTS

| **Questions** | **Score** | **Before EPNS** | **After 2 weeks of EPNS** | **After 4 weeks of EPNS** | **After 8 weeks of EPNS** |
| --- | --- | --- | --- | --- | --- |
| **1a. Is there a delay before you can start to urinate?** |  |  |  |  |  |
| Never | 0 |  |  |  |  |
| Occasionally | 1 |  |  | √ | √ |
| Sometimes | 2 |  |  |  |  |
| Most of the time | 3 |  | √ |  |  |
| All of the time | 4 | √ |  |  |  |
| **1b. How much does this bother you?** | 0-10 | 10 | 9 | 5 | 4 |
| **2a. Do you have to strain to continue urinating?** |  |  |  |  |  |
| Never | 0 |  |  |  |  |
| Occasionally | 1 |  |  |  |  |
| Sometimes | 2 |  |  | √ | √ |
| Most of the time | 3 |  | √ |  |  |
| All of the time | 4 | √ |  |  |  |
| **2b. How much does this bother you?** | 0-10 | 10 | 8 | 5 | 5 |
| **3a. Would you say that the strength of your urinary stream is…** |  |  |  |  |  |
|  |  |  |  |  |  |
| Normal | 0 |  |  |  |  |
| Occasionally reduced | 1 |  |  |  |  |
| Sometimes reduced | 2 |  |  | √ | √ |
| Reduced most of the time | 3 |  | √ |  |  |
| Reduced all of the time | 4 | √ |  |  |  |
| **3b. How much does this bother you?** | 0-10 | 8 | 7 | 4 | 4 |
| **4a. Do you stop and start more than once while you urinate？** |  |  |  |  |  |
| Never | 0 |  |  |  |  |
| Occasionally | 1 |  |  |  |  |
| Sometimes | 2 |  |  | √ | √ |
| Most of the time | 3 |  | √ |  |  |
| All of the time | 4 | √ |  |  |  |
| **4b. How much does this bother you?** | 0-10 | 8 | 8 | 4 | 4 |
| **5a. How often do you feel that your bladder has not emptied properly after you have urinated?** |  |  |  |  |  |
| Never | 0 |  |  |  |  |
| Occasionally | 1 |  |  |  |  |
| Sometimes | 2 |  |  | √ | √ |
| Most of the time | 3 |  | √ |  |  |
| All of the time | 4 | √ |  |  |  |
| **5b. How much does this bother you?** | 0-10 | 10 | 8 | 5 |  |
| **6a. Do you have a sudden need to rush to the toilet to urinate ?** |  |  |  |  |  |
| Never | 0 |  |  | √ | √ |
| Occasionally | 1 |  | √ |  |  |
| Sometimes | 2 | √ |  |  |  |
| Most of the time | 3 |  |  |  |  |
| All of the time | 4 |  |  |  |  |
| **6b. How much does this bother you?** | 0-10 | 6 | 4 | 0 | 0 |
| **7a. Does urine leak before you can get to the toilet？** |  |  |  |  |  |
| Never | 0 | √ | √ | √ | √ |
| Occasionally | 1 |  |  |  |  |
| Sometimes | 2 |  |  |  |  |
| Most of the time | 3 |  |  |  |  |
| All of the time | 4 |  |  |  |  |
| **7b. How much does this bother you?** | 0-10 | 0 | 0 | 0 | 0 |
| **8a. Does urine leak when you cough or sneeze？** |  |  |  |  |  |
| Never | 0 | √ | √ | √ | √ |
| Occasionally | 1 |  |  |  |  |
| Sometimes | 2 |  |  |  |  |
| Most of the time | 3 |  |  |  |  |
| All of the time | 4 |  |  |  |  |
| **8b. How much does this bother you?** | 0-10 | 0 | 0 | 0 | 0 |
| **9a. Do you ever leak for no obvious reason and without feeling that you want to go?** |  |  |  |  |  |
| Never | 0 | √ | √ | √ | √ |
| Occasionally | 1 |  |  |  |  |
| Sometimes | 2 |  |  |  |  |
| Most of the time | 3 |  |  |  |  |
| All of the time | 4 |  |  |  |  |
| **9b. How much does this bother you?** | 0-10 | 0 | 0 | 0 | 0 |
| **10a. Do you leak urine when you are asleep?** |  |  |  |  |  |
| Never | 0 | √ | √ | √ | √ |
| Occasionally | 1 |  |  |  |  |
| Sometimes | 2 |  |  |  |  |
| Most of the time | 3 |  |  |  |  |
| All of the time | 4 |  |  |  |  |
| **10b. How much does this bother you?** | 0-10 | 0 | 0 | 0 | 0 |
| **11a. How often have you had a slight wetting of your pants a few minutes after you had finished urinating and had dressed yourself?** |  |  |  |  |  |
| Never | 0 | √ | √ | √ | √ |
| Occasionally | 1 |  |  |  |  |
| Sometimes | 2 |  |  |  |  |
| Most of the time | 3 |  |  |  |  |
| All of the time | 4 |  |  |  |  |
| **11b. How much does this bother you?** | 0-10 | 0 | 0 | 0 | 0 |
| **12a. How often do you pass urine during the day？** |  |  |  |  |  |
| 1 to 6 times | 0 |  |  |  |  |
| 7 to 8 times | 1 |  |  |  | √ |
| 9 to 10 times | 2 |  |  | √ |  |
| 11 to 12 times | 3 |  | √ |  |  |
| 13 or more times | 4 | √ |  |  |  |
| **12b. How much does this bother you?** | 0-10 | 8 | 8 | 4 | 2 |
| **13a. During the night，how many times do you have to get up to urinate, on average?** |  |  |  |  |  |
| None | 0 |  |  |  |  |
| One | 1 |  |  |  |  |
| Two | 2 |  |  | √ | √ |
| Three | 3 |  | √ |  |  |
| Four or more | 4 | √ |  |  |  |
| **13b. How much does this bother you?** | 0-10 | 10 | 8 | 5 | 4 |
| **Total score** |  | 30 | 22 | 14 | 13 |

ICIQ-MLUTS: International Consultation on Incontinence Questionnaire - Male Lower Urinary Tract Symptoms
